# Pharmacology of Rasagiline, a New MAO-B Inhibitor Drug for the Treatment of Parkinson’s Disease with Neuroprotective Potential

**DOI:** 10.5041/RMMJ.10003

**Published:** 2010-07-02

**Authors:** John P.M. Finberg

**Affiliations:** Departments of Molecular Pharmacology, The Bruce Rappaport Faculty of Medicine, and the Rappaport Institute for Research in the Medical Sciences, Technion-Israel Institute of Technology, Haifa 31096, Israel

**Keywords:** monoamine oxidase, dopamine, Parkinson’s disease, neuroprotection, cheese effect

## Abstract

Rasagiline (Azilect) is a highly selective and potent propargylamine inhibitor of monoamine oxidase (MAO) type B. Like other similar propargylamine inhibitors, rasagiline binds covalently to the N5 nitrogen of the flavin residue of MAO, resulting in irreversible inactivation of the enzyme. Therapeutic doses of the drug which inhibit brain MAO-B by 95% or more cause minimal inhibition of MAO-A, and do not potentiate the pressor or other pharmacological effects of tyramine. Metabolic conversion of the compound *in vivo* is by hepatic cytochrome P450-1A2, with generation of 1-aminoindan as the major metabolite. Rasagiline possesses no amphetamine-like properties, by contrast with the related compound selegiline (Deprenyl, Jumex, Eldepryl). Although the exact distribution of MAO isoforms in different neurons and tissues is not known, dopamine behaves largely as a MAO-A substrate *in vivo*, but following loss of dopaminergic axonal varicosities from the striatum, metabolism by glial MAO-B becomes increasingly important. Following subchronic administration to normal rats, rasagiline increases levels of dopamine in striatal microdialysate, possibly by the build-up of β-phenylethylamine, which is an excellent substrate for MAO-B, and is an effective inhibitor of the plasma membrane dopamine transporter (DAT). Both of these mechanisms may participate in the anti-Parkinsonian effect of rasagiline in humans. Rasagiline possesses neuroprotective properties in a variety of primary neuronal preparations and neuron-like cell lines, which is not due to MAO inhibition. Recent clinical studies have also demonstrated possible neuroprotective properties of the drug in human Parkinsonian patients, as shown by a reduced rate of decline of symptoms over time.

## INTRODUCTION

The most important and debilitating symptoms of Parkinson’s disease are those resulting from dopamine (DA) depletion in the nigro-striatal pathway, although the disease process starts at an earlier stage, and is marked by symptoms such as reduced olfactory sensitivity, autonomic dysfunction, and affective disorder.^1^ All currently available treatments are symptomatic (Table 1), despite a large effort to find new drugs which can counteract the accelerated rate of neuronal loss, and a major research effort to understand the mechanisms involved in neurodegeneration. The original “DA replacement” therapy, i.e. 3,4-dihydroxy-phenylalanine (L-dopa), remains the most effective symptomatic treatment, although its use is accompanied by serious motor, psychiatric and other side effects.^2^–^4^ Additional therapies which increase the availability of DA at its striatal receptor sites include monoamine oxidase type B (MAO-B) inhibitors and catechol O-methyl transferase (COMT) inhibitors, as well as surgical procedures such as transplantation of DA-releasing cells. Direct DA agonists are also extensively used.^5^ The full therapeutic arsenal includes anti-muscarinic drugs, amantadine derivatives, and focal stimulation of appropriate basal ganglia nuclei such as the sub-thalamic nucleus. The current article describes the new, selective propargyl MAO-B inhibitor rasagiline, developed by the author together with Professor Moussa Youdim at the Rappaport Faculty of Medicine, Technion, Haifa, Israel, in conjunction with Teva Pharmaceuticals, Israel.

Rasagiline exerts its anti-Parkinsonian effect by inhibiting the oxidative breakdown of DA in the striatum, and can be used both as monotherapy in the early stages of the disease, or as an adjunct to L-dopa, in the advanced stages.^6^,^7^ An additional property of rasagiline, however, is its neuroprotective effect, which in preclinical studies was shown to be independent of its MAO-inhibitory effect, and has recently been found to occur in patients treated with normal therapeutic doses of the drug, using an innovative clinical trial with delayed start design.^8^,^9^ This recent finding in patients provides an interesting example of “bench to bedside” in action, although the mechanism of rasagiline’s putative neuroprotective effect in patients is at present not clear.

## MONOAMINE OXIDASE INHIBITORS AS ANTI-PARKINSONIAN DRUGS

The enzyme MAO is responsible for the oxidative deamination of a wide range of biogenic and xenobiotic amines, including DA, noradrenaline, adrenaline, tyramine, serotonin, β-phenylethylamine, N-methylhistamine, benzylamine, and methoxy metabolites of the parent amines, such as metanephrine and normetanephrine^10^ (Table 2). Being situated within axonal varicosities, it plays a major role in the oxidative metabolism of the major monoamine neurotransmitters, i.e. noradrenaline, serotonin (5-HT) and DA.

The first clinical use of MAO inhibitors was in the treatment of depressive disease, an effect mediated by inhibition of the degradation of noradrenaline and 5-HT, and consequent increased levels of these amines at their receptors. In the Parkinsonian patient, in whom DA levels are reduced, inhibition of DA oxidative metabolism can also be effective in returning neurotransmitter levels towards normal; however, non-selective inhibition of MAO can cause dangerous increases in amine levels, especially in conjunction with a monoamine precursor such as L-dopa or indirectly acting amine such as tyramine. Following the introduction of the selective inhibitors clorgyline and selegiline, together with biochemical experiments which succeeded in separating different isoforms of the enzyme, MAO was shown to exist in two isoforms known as MAO-A and MAO-B, which show different selectivities for substrates and inhibitors^11^ (Table 2). An important aspect of the existence of the two isoforms is their cellular localization, since the two isoforms are expressed in different cells and tissues (Table 2). The enzyme is located intracellularly, inserted in the outer mitochondrial membrane, with its active site in the cytoplasmic space.^12^ Type A MAO shows highest affinity for hydroxylated amines such as noradrenaline and 5-HT, while type B MAO has greatest affinity for non-hydroxylated amines such as β-phenylethylamine and benzylamine. Some amines, notably DA and tyramine, have equal affinity for both enzyme isoforms.

The enzyme MAO is widely distributed in the body’s tissues, with a high degree of expression in the gastro-intestinal tract and liver, as well as neuronal tissue, and also in lung, heart, placenta, and nearly all other organs. For the current discussion, however, the most important aspect of the selective distribution of MAO-A and MAO-B is their selective expression in neurons and cells of the nervous system. Within neurons, MAO enzyme molecules are synthesized in the perikaryon, and inserted into the mitochondrial outer membrane. Mitochondria are transported to the axon terminals by axonal transport. Little is known about the way in which MAO subtypes are trafficked along the axons, but this process may well be important in determining the subtype expressed in axonal varicosities. The selective occurrence of MAO subtypes in neuronal and glial tissue is an important factor in understanding how subtype-selective MAO inhibitors may affect synaptic neurotransmitter levels.

Sympathetic denervation studies showed that MAO-A is the predominant subtype in sympathetic post-ganglionic neurons.^13^ Because of technical difficulties, the question of subtype distribution within CNS neurons is still not completely resolved, the difficulty being the small size of axon terminals. Using techniques of *in situ* hybridization and immunohistochemistry, MAO-A has been localized to noradrenergic perikarya of locus coeruleus, while MAO-B was the predominant subtype expressed in serotonergic cell bodies of the raphe nucleus and in glial cells.^14^–^19^ These findings were similar in rodent and primate species; however, in rats, production of oxidized metabolites of catecholamines and 5-HT was reduced by inhibitors of MAO-A but not by inhibitors of MAO-B, showing that these neurotransmitters are substrates of MAO-A *in vivo*.^20^ A possible explanation for this phenomenon is that different populations of mitochondria may express different MAO subtypes, and axonal transport of one subtype or the other may lead to selective occurrence of MAO-A in axon terminals of both serotonergic and noradrenergic neurons.^21^ According to this concept, the neurotransmitter molecules are mainly taken up into the axon terminals following release to the synaptic space and metabolized by the MAO type in the axonal varicosities (i.e. MAO-A), even though the cell bodies may contain the opposite subtype. In the case of DA, the form of MAO expressed in axonal varicosities of dopaminergic neurons is thought to be MAO-A, since in rodents, inhibitors of MAO-A cause marked increases in extracellular and tissue levels of DA, whereas MAO-B inhibitors have little effect.^22^,^23^ On the other hand, in primate brain, MAO-B levels are considerably higher than those of MAO-A, possibly because glial MAO is largely of the MAO-B subtype and DA may well be partially taken up by glial cells after its physiological release from neurons, and deaminated within the glia. In support of this hypothesis, rasagiline was found to increase extracellular DA levels in normal monkey brain after systemic administration of L-dopa.^24^

The breakdown of monamines by MAO can be described by the equation:

$$\text{R}-{\text{CH}}_{2}-{\text{NH}}_{2}+{\text{O}}_{2}+{\text{H}}_{2}\text{O}\overset{\text{MAO}}{\to }\text{R}-\text{CHO}+{\text{NH}}_{3}+{\text{H}}_{2}{\text{O}}_{2}$$

Several important facts are contained within this expression, including the dependence on free oxygen, the initial production of an aldehyde, and the release of hydrogen peroxide as well as ammonia following deamination of the substrate. The aldehydes are metabolized by the aldehyde metabolizing enzymes, aldehyde reductase and aldehyde dehydrogenase, to carboxylic acids and glycols, and hydrogen peroxide is converted by catalase to water and oxygen. In the case of dysfunction of such effective scavenger enzymes, however, potentially damaging reactive aldehydes and free radicals may be generated.

## DEVELOPMENT OF RASAGILINE

**Figure 1.**
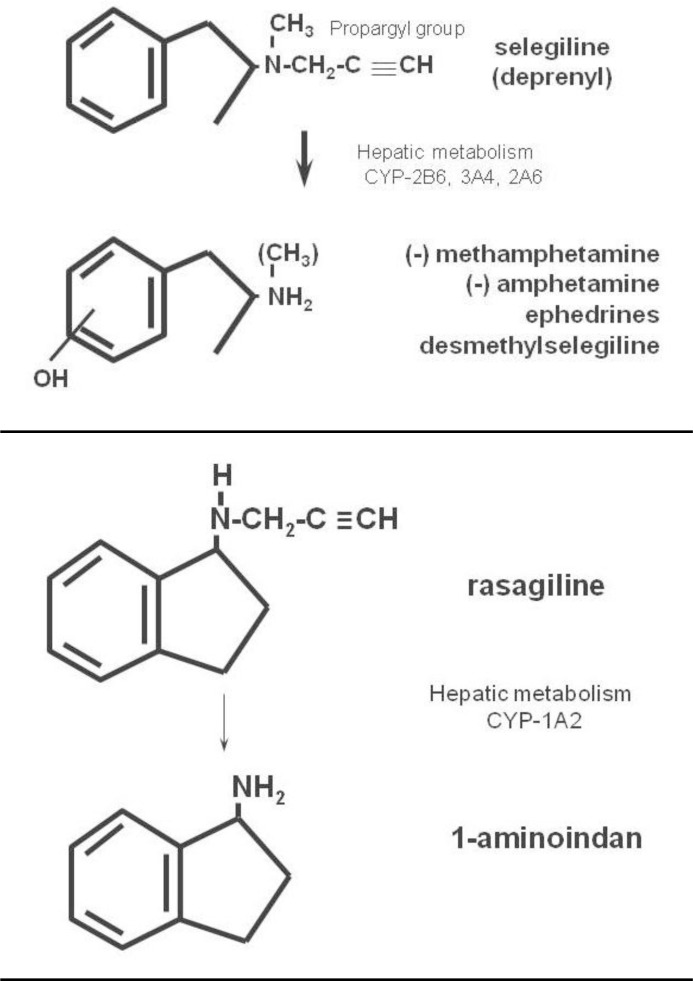
Structures of selegiline, rasagiline, and their metabolites.

The first selective MAO-B inhibitor to be described was selegiline, which was synthesized in 1965 by Knoll and Magyar, based on methamphetamine with the addition of a propargyl group (Figure 1).^25^ Following administration, selegiline is extensively metabolized by hepatic cytochrome P450 2A6, 2B6, and 3A4 with the production of methamphetamine and a small percentage of other metabolites. Since selegiline is of the R(−) configuration, R(−)-methamphetamine is formed (in older nomenclature, L[−]-methamphetamine). This enantiomer of methamphetamine is often erroneously stated to be pharmacologically inactive. In fact, although S(+)-methamphetamine (previously D[+]-methamphetamine) possesses greater CNS behavioral activity, the two enantiomers have similar potency for inhibition of the plasma membrane noradrenaline transporter (NET).^26^

In a large number of *in vitro* and *in vivo* pharmacological tests, selegiline was shown not to potentiate the actions of tyramine, while at the same time potentiating those of β-phenylethylamine.^25^ This finding was interpreted by Knoll et al.^25^ as showing that selegiline possesses NET-inhibitory activity as well as MAO-inhibitory activity, since inhibition of uptake inhibits the action of indirectly acting sympathomimetic amines such as tyramine. The potentiation of phenylethylamine’s effect was thought to be caused by greatly reduced metabolism of this amine. In fact, selegiline itself possesses only weak uptake-inhibitory activity.^27^

Selegiline was introduced into clinical medicine for treatment of Parkinson’s disease by Birkmayer and associates.^28^,^29^ Following early preclinical studies showing that it enhanced the life span of laboratory rats,^30^ selegiline was found to reduce death rate in human patients with Parkinson’s disease, but this could be due to improved clinical status of the patients rather than a true neuroprotective effect.^31^ Selegiline has also been found to reduce cell death in neuronal cell line types, such as PC-12 and SH-SY5Y.^32^,^33^ Following on these findings, the Parkinson’s Disease Study Group arranged a large multicenter clinical trial to determine whether selegiline, alone or in combination with alpha-tocopherol, reduces the rate of progression of the disease (DATATOP study). This trial showed that selegiline alone possesses significant symptomatic effect, but could not distinguish this from true neuroprotective effect, because the symptomatic effect of selegiline masked possible underlying disease progression.

Rasagiline is a close chemical relative of selegiline, but possesses the important distinction that its major metabolite is 1-aminoindan, which does not possess amphetamine-like activity, and does not possess appreciable affinity for any of the catecholaminergic or serotonergic receptor groups.^20^,^34^,^35^ Rasagiline possesses a similar degree of selectivity to selegiline for inhibition of MAO-B as compared with MAO-A,^36^ in rat hepatic and brain tissue both *in vivo* and *in vitro*, but is significantly more potent than selegiline, both in rat and man. Both inhibitors will inhibit the A form of the enzyme at higher doses.

The propargyl derivative inhibitors are irreversible site-directed inhibitors, which form covalent linkage with the N5 nitrogen of flavin, a component of the enzyme active site. When used clinically, the drugs are administered at a low daily dose, which inhibits a small fraction of the enzyme at each administration. The degree of enzyme inhibition thereby increases with successive doses of the inhibitor. The aim is to use a daily dose at which nearly complete inhibition of the enzyme occurs after about 10 days, so that subsequent drug administration maintains the extensive inhibition of the enzyme by inhibiting newly synthesized enzyme.

Rasagiline is mainly metabolized by the hepatic cytochrome P450 enzyme 1A2, with production of 1-aminoindan as the major metabolite.^37^, ^38^

### RASAGILINE AND THE “CHEESE EFFECT”

The advent of rasagiline enabled confirmation of the hypothesis that tyramine potentiation results from inhibition of MAO-A but not MAO-B. This point was extensively studied by us in pharmacological experiments using the rat vas deferens preparation *in vitro*.^39^,^40^ Vas deferens contains an extremely dense sympathetic innervation, and the tissue contracts following sympathetic nerve stimulation, or addition of α_1_-adrenoceptor agonists. By combining biochemical determination of tissue MAO activities with pharmacological response to tyramine and noradrenaline, we were able to show that tyramine potentiation occurred following 80% or more inhibition of MAO-A, but not of MAO-B.^40^

**Figure 2.**
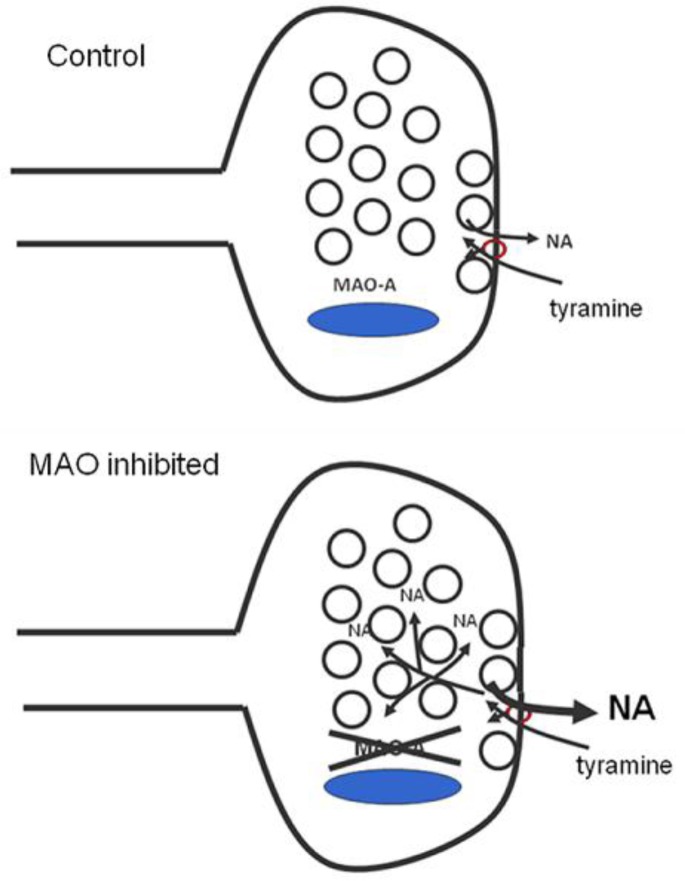
Mechanism of potentiation of tyramine effects by monoamine oxidase (MAO) inhibition. In the control situation (above) tyramine is transported into axon terminal by the noradrenaline transporter (NET) and noradrenaline (NA) is transported out, but few Na molecules are available in the soluble cytoplasmatic pool because of the activity of intraneuronal MAO-A. Following MAO inhibition (below) the size of the soluble NA pool increases resulting in a much greater chance for reverse transport. Note that some NA is also displaced from the granular storage pool by tyramine.

At the whole animal level, cheese effect can theoretically result from a decrease in breakdown of orally administered tyramine in intestinal tract and liver, tissues which both express large amounts of both subtypes of the MAO enzyme. Our work with the isolated tissue preparation, however, showed that an important part of the cheese effect is potentiation of tyramine’s ability to release noradrenaline at the level of the neuron. Selective inhibition of MAO within the sympathetic neuron could be working in two ways: either by increasing the level of tyramine within the neuron, or by increasing the cytoplasmatic level of noradrenaline available for release. The latter would appear to be the most significant mechanism. Release of noradrenaline by tyramine is non-exocytotic. As shown by Trendelenburg and associates,^41^ all indirectly-acting amines are substrates for NET. Transport of the indirectly acting amine brings the active site of the transporter to the internal side of the membrane, where any available noradrenaline molecules will be able to combine with it and be transported out of the neuron and into the synaptic cleft (Figure 2).

## ACTION OF RASAGILINE ON DOPAMIN RELEASE

During my sabbatical studies at NIH, Bethesda, Maryland, in the laboratories of Drs I. Kopin, D. Goldstein, and K. Bankiewycz, I used the micro-dialysis technique to study the metabolism of DA in rat striatum which had been depleted of dopaminergic innervation by local application of the neurotoxin 6-hydroxydopamine to the substantia nigra. These studies showed that MAO-A is the dominant enzyme subtype in the metabolism of DA in rat striatum, both in intact striatum, and following loss of dopaminergic input.^22^ Similar findings were reported by Wachtel and Abercrombie.^23^ In our subsequent studies with rasagiline at Haifa, however, we showed that when administered over a period of about 2 weeks to normal, non-lesioned rats, low, selective doses of the MAO-B inhibitors increased striatal extracellular fluid levels of DA.^42^ The explanation for this phenomenon may be the accumulation of β-phenylethylamine in brain tissue following the long-term treatment. This amine is an indirectly acting releaser of DA, which is continually produced from phenylalanine but normally is rapidly metabolized by MAO-B. Chronic treatment with MAO-B inhibitors may therefore lead to accumulation of β-phenylethylamine and non-exocytotic release of DA, by a similar mechanism to that whereby tyramine releases noradrenaline from sympathetic nerves. Accumulation of β-phenylethylamine following MAO-B inhibition was demonstrated by Boulton and coworkers.^43^,^44^ Although β-phenylethylamine may be involved in release of DA from intact dopaminergic nerve fibers (and/or inhibition of its reuptake), in the advanced Parkinsonian brain, physiological DA release in the striatum is largely absent, and the phenylethylamine mechanism will not be effective, although post-synaptic effects of phenylethylamine have also been detected.^45^ Another aspect of the effect of MAO-B inhibitors which is important in the Parkinsonian brain is their ability to enhance striatal DA levels following administration of systemic L-dopa. When a significant number of dopaminergic nerves are still present, L-dopa is taken up by these neurons and converted to DA in a single decarboxylation by the enzyme aromatic amino acid decarboxylase (AAAD). This enzyme is quite ubiquitous, occurring in many cell types in the CNS, including serotonergic neurons, glial cells, and other types of neurons apart from the dopaminergic neurons. In advanced Parkinson’s disease, when 95% or even more of the nigro-striatal dopaminergic neurons are missing, the important neurons involved in L-dopa decarboxylation become the serotonergic ones, because striatum receives a dense serotonergic innervation. Studies in the rat have shown that loss of striatal DA innervation is followed by a compensatory increase in serotonergic innervation.^46^ This may also be the situation at some stage of Parkinson’s disease; however, in the advanced Parkinsonian brain, the serotonergic midbrain raphe nucleus, from which the striatal fibers originate, also degenerates and is lost. We are currently studying where L-dopa is deaminated following loss of both dopaminergic and serotonergic axonal varicosities. Our preliminary data show that following both dopaminergic denervation by 6-hydroxydopamine, and 5-HT depletion by 5,6-dihydroxytryptamine, rasagiline treatment causes a greater increase in DA produced from L-dopa than following single lesion with 6-hydroxydopamine alone.^47^ It is conceivable that a greater proportion of administered L-dopa is decarboxylated to DA in glial cells (which express MAO-B) in the Parkinsonian brain. Rasagiline and other MAO-B inhibitors may therefore produce some of their clinical L-dopa potentiating effect by inhibition of glial MAO-B. In the early-stage Parkinsonian brain, where a substantial number of DA neurons are still present, the β-phenylethylamine mechanism may participate in the anti-Parkinsonian effect of rasagiline.

## RASAGILINE AND NEUROPROTECTION

### NEUROPROTECTION IN ANIMAL MODELS AND CELLS

As described above, selegiline was found to possess neuroprotective effect in isolated neuronal preparations. Neuroprotection was observed at concentrations below those required for MAO inhibition, and other MAO inhibitors did not consistently produce neuroprotection. We observed neuroprotection by rasagiline both in dopaminergic and non-dopaminergic rat embryonic mesencephalic neurons.^48^ The neuroprotective effect of rasagiline was greater than that of selegiline at equimolar concentrations, although this action was seen at a relatively high concentration of rasagiline (1 μM). Later, we described the anti-apoptotic action of rasagiline in primary cultures of rat cerebellar neurons, which are non-catecholaminergic.^49^ The neuroprotective effect in cerebellar granule cells was seen at concentrations (1 X 10^–10^ M) below those required for MAO inhibition (1 X 10^–8^M).

The intracellular mechanism of action of rasagiline’s anti-apoptotic effect has been extensively investigated by Youdim and co-workers.^50^–^53^ The proposed mechanisms of action include up-regulation of Bcl2 family proteins, reduced expression of pro-apoptotic Bax family proteins, up-regulation of protein kinase C ε, up-regulation of superoxide dismutase, and antagonism of nuclear translocation of glyceraldehyde phosphate dehydrogenase (GAPDH). Most, but not all of these effects have been described at therapeutically relevant concentrations (nanomolar). It is most interesting that so many anti-apoptotic actions are possessed by a single molecule, which raises the possibility of a receptor site for this molecule. A binding site for selegiline was found on the GAPDH molecule by Tatton and coworkers,^54^ but the existence of a similar site for rasagiline has not yet been established.

At the whole animal level, rasagiline has been observed to reverse MPTP-induced reduction of tyrosine hydroxylase-positive neurons in the substantia nigra in mice as well as the neurological deficit caused by the MPTP administration.^53^ Since neurogenesis does not occur in mouse substantia nigra, rasagiline must therefore enhance the expression of tyrosine hydroxylase which has been down-regulated by the neurotoxin. The important aspect of this study is that rasagiline administration was commenced 2 weeks after MPTP had been given and tyrosine hydroxylase levels had already been depleted.

### CLINICAL STUDIES WITH RASAGILINE

Clinical anti-Parkinsonian effect of rasagiline was described in two major clinical trials. The first (TEMPO)^6^ compared rasagiline with entacapone (peripherally-acting COMT inhibitor). Both drugs were found to cause significant anti-Parkinsonian effect as shown by reduction of about 2 points in the UPDRS clinical rating scale. The second (LARGO)^7^ was designed to establish the efficacy of rasagiline in combination with L-dopa. In this study rasagiline was effective both in increasing “on” time and reducing the severity of “off”. Building on the experience with DATATOP and other studies, rasagiline was chosen by the Parkinson’s Study Group for a new trial (ADAGIO)^8^,^9^ designed to detect whether the drug can reduce disease progression. Since the estimation of disease protection is made on the basis of clinical neurological score (UPDS), any symptomatic drug will give a false positive result. The test format adopted in ADAGIO was to compare drug and placebo groups of recent onset patients for a period of time judged to be sufficient for detectable disease progression (9 months) and then put all patients on the active drug therapy for an additional period of 9 months. At the end of this period, patients were compared for clinical status. Since all patients received active drug therapy at conclusion of the trial, the symptomatic effect of the drug was balanced out. It was found that patients who received the drug at 1 mg daily for 18 months finished the trial period in a significantly better clinical status than those who received it for only 9 months, although this effect was not significant at a dose of 2 mg.

## SUMMARY

Rasagiline is a selective MAO-B inhibitor which is devoid of the amphetamine-like actions of its predecessor, selegiline. The drug has two distinct actions: selective irreversible inhibition of MAO-B, and neuroprotective effect not dependent on MAO inhibition. When used in patients, the possibility exists that its action to retard disease progression is linked to MAO inhibition, since activation of DA receptors can induce release of a variety of neurotrophic factors, but reduced disease progression could also be an expression of the neuroprotective action seen in laboratory experiments. Further understanding of the mechanism of clinical action in retardation of disease progression will require development of better techniques to understand the consequences of inhibition of MAO-B in the human brain.

## Figures and Tables

**Table 1.** Drugs used in treatment of Parkinson’s disease.

Dopamine replacement therapy	L-dopa + peripherally acting decarboxylase inhibitor (carbidopa, benserazide)
Direct dopamine agonists	Ropinirole, rotigotine, bromocriptine, pergolide, cabergoline, pramipexole, apomorphine
MAO-B inhibitors	Rasagiline, selegiline
COMT inhibitors	Entacapone*, tolcapone**†**
Antimuscarinic, DAT inhibitor	Benztropine**‡**, trihexiphenidyl
NMDA receptor antagonists§	Amantadine, memantine

MAO denotes monoamine oxidase, DA dopamine, DAT plasma membrane dopamine transporter.NET plasma membrane noradrenaline transporter, COMT catechol O-methyl transferase, and L-dopa, 3,4-dihydroxyphenylalanine.

* Peripherally acting inhibitor.

† Peripherally + centrally acting inhibitor.

‡ Also inhibits DA uptake via plasma membrane DA transporter.

§ Glutamate antagonists, moderate efficacy in Parkinsonism and L-dopa-induced dyskinesias.

**Table 2.** Monoamine oxidase (MAO) subtypes, their substrates and inhibitors and cellular localization.

	**MAO-A**	**MAO-B**
Selective inhibitors (irreversible, covalent combination with enzyme active site)	Clorgyline	Selegiline (deprenyl), rasagiline
Selective inhibitors (reversible, competitive inhibition)	Moclobemide	Lazabemide
Non-selective inhibitors	Phenelzine, tranylcypromine	
Selective substrates	5-HT, noradrenaline, adrenaline	β-phenylethylamine, benzylamine
Mixed substrates	Dopamine, tyramine	
Cellular localization	Sympathetic neurons, noradrenergic, dopaminergic and other neurons in CNS, placenta, GI tract, hepatocytes and many other cell types	Astrocytes, platelets, many other peripheral cell types, serotonergic neuronal cell bodies in raphe nuclei

## Abbreviations:

MAO: monoamine oxidase;
DA: dopamine;
DAT: plasma membrane dopamine transporter;
NET: plasma membrane noradrenaline transporter;
COMT: catechol O-methyl transferase;
L-dopa: 3,4-dihydroxyphenylalanine.
